# Optimizing a Whole-Genome Sequencing Data Processing Pipeline for Precision Surveillance of Health Care-Associated Infections

**DOI:** 10.3390/microorganisms7100388

**Published:** 2019-09-24

**Authors:** Weihua Huang, Guiqing Wang, Changhong Yin, Donald Chen, Abhay Dhand, Melissa Chanza, Nevenka Dimitrova, John T. Fallon

**Affiliations:** 1 Department of Pathology, New York Medical College, Valhalla, NY 10595, USA; Hank.Wang@wmchealth.org (G.W.); changhong_yin@nymc.edu (C.Y.); mchanza@nymc.edu (M.C.); John.Fallon@wmchealth.org (J.T.F.); 2 Department of Pathology and Clinical Laboratories, Westchester Medical Center, Valhalla, NY 10595, USA; 3 Department of Medicine, New York Medical College, Valhalla, NY 10595, USA; Donald.Chen@wmchealth.org (D.C.); Abhay.Dhand@wmchealth.org (A.D.); 4 Department of Infection Prevention and Control, Westchester Medical Center, Valhalla, NY 10595, USA; 5 Philips Research North America, Cambridge, MA 02141, USA; Nevenka.Dimitrova@philips.com

**Keywords:** health care-associated infection (HAI), genomic surveillance, whole-genome sequencing (WGS), k-mer, data processing pipeline

## Abstract

The surveillance of health care-associated infection (HAI) is an essential element of the infection control program. While whole-genome sequencing (WGS) has widely been adopted for genomic surveillance, its data processing remains to be improved. Here, we propose a three-level data processing pipeline for the precision genomic surveillance of microorganisms without prior knowledge: species identification, multi-locus sequence typing (MLST), and sub-MLST clustering. The former two are closely connected to what have widely been used in current clinical microbiology laboratories, whereas the latter one provides significantly improved resolution and accuracy in genomic surveillance. Comparing to a broadly used reference-dependent alignment/mapping method and an annotation-dependent pan-/core-genome analysis, we implemented our reference- and annotation-independent, k-mer-based, simplified workflow to a collection of *Acinetobacter* and *Enterococcus* clinical isolates for tests. By taking both single nucleotide variants and genomic structural changes into account, the optimized k-mer-based pipeline demonstrated a global view of bacterial population structure in a rapid manner and discriminated the relatedness between bacterial isolates in more detail and precision. The newly developed WGS data processing pipeline would facilitate WGS application to the precision genomic surveillance of HAI. In addition, the results from such a WGS-based analysis would be useful for the precision laboratory diagnosis of infectious microorganisms.

## 1. Introduction

Health care-associated infection (HAI) is a significant cause of illness and death, continuing to threaten the health care system. The Centers for Disease Control and Prevention (CDC) in the United States estimates (https://www.cdc.gov/hai/data/index.html) that approximately one in 31 hospital patients has at least one HAI every day, although significant progress has been made in prevention and control. These nosocomial infections lead to the loss of tens of thousands of lives and pose a significant cost burden each year, millions to billions of dollars [1]. By providing solid data-driven evidence to accurately identify infected patients, determine the infection factors and sites, guide appropriate intervention measures, and further evaluate the intervention efficacy, precision surveillance is an essential element of improving infection control. 

In the past decade, advances in molecular techniques have created a new gold standard for rapid and accurate bacterial identification in clinical microbiology laboratories, such as multi-locus sequence typing (MLST) [2], 16S ribosomal RNA (rRNA) target sequencing [3], and matrix-assisted laser desorption/ionization time-of-flight mass spectrometry (MALDI-TOF MS) [4]. In addition, recent advances in next-generation sequencing (NGS) have demonstrated its sequence-evidenced superior discriminatory power. The high resolution (up to single base) of whole-genome sequencing (WGS) allows precision microbial identification and characterization for accurate outbreak investigations, phylogeographic distribution, and/or evolutionary studies, facilitating a better understanding of infectious pathogens. Genomic surveillance or genomic epidemiology has thus become a key weapon in fighting against microbial pathogens [5,6].

In this study, we developed, simplified, and optimized a WGS data processing pipeline for the precision surveillance of bacterial infections. To demonstrate its application, we used a collection of *Acinetobacter baumannii* and some rare species of vancomycin-resistant enterococci (VRE) clinical isolates. Both VRE and *Acinetobacter* bacteria are recognized as critical major nosocomial pathogens (https://www.cdc.gov/hai/data/index.html) due to their natural intrinsic resistance to several antimicrobials and capacity to quickly acquire virulence and multidrug resistance. 

## 2. Materials and Methods

The Institutional Review Board of New York Medical College approved this study.

### 2.1. Collection of Bacterial Isolates

All bacterial isolates were recovered from patients, except one from environment surveillance (M170981), at the Clinical Microbiology Laboratory of Westchester Medical Center (WMC), a tertiary-care hospital in suburban New York City. Along with culture and identification by routine laboratory tests, these bacterial isolates were verified using the MALDI Biotyper CA system (Bruker, Billerica, MA, USA) and subjected to the MicroScan WalkAway automated system (Beckman Coulter, Brea, CA, USA) for antimicrobial susceptibility tests. During our WGS clinical surveillance of multidrug-resistant bacteria from November 2016 to the end of 2018, a total of 147 *Acinetobacter* sp. isolates were collected from 34 patients and one environmental swab. A local *A. baumannii* clinical isolate PB364 was collected in 2016 from a patient too and subjected to whole genome assembly with both short- and long-read NGS—the genome of which was used as a reference in data analysis. Five vancomycin-resistant *Enterococcus* sp. clinical isolates were collected in February 2019. When hospital-associated VRE transmission was suspected, they were compared to two *E. gallinarum* clinical isolates also collected in February 2019 and four *Enterococcus* sp. clinical isolates in stock collected previously, from 2013 to 2018.

### 2.2. Next-Generation Sequencing (NGS)

DNA was extracted from bacterial isolates using the Agencourt GenFind DNA Isolation Kit and the Biomek FX^P^ Automated Workstation (Beckman Coulter). A short-read NGS library was prepared using the Nextera DNA Flex or XT Library Prep Kit (Illumina, San Diego, CA, USA). DNA quantification was performed in a microplate using the SpectraMax Quant dsDNA Assay Kit and the Gemini XPS Fluorometer (Molecular Devices, San Jose, CA, USA), or in a single tube using the Qubit 2.0 fluorometer (Thermo Fisher Scientific, Springfield Township, NJ, USA). Library quality was examined using the 4200 TapeStation and D1000 ScreenTape (Agilent, Santa Clara, CA, USA). Paired-end sequencing (2 × 150 cycles) was performed using the NextSeq 550 system (Illumina). The Pacific Biosciences (PacBio, Menlo Park, CA, USA) RSII single-molecule real-time (SMRT) sequencing system was employed for the long-read sequencing of *A. baumannii* PB364. The long-read NGS library was processed using g-TUBE fragmentation (Covaris, Woburn, MA, USA), BluePippin size selection (Sage Science, Beverly, MA, USA) and a SMRTbell template preparation kit (PacBio). Mainly, the manufacturers’ standard protocols were followed.

### 2.3. Bioinformatics Analysis

Multiple free-source algorithms were employed in the optimization of workflows, which included: trimmomatic v0.35 [7] for sequence trimming and cleaning; SPAdes [8] for de novo genome assembling; Kraken v0.10.5-beta (updated to Kraken2 later) [9] for taxonomy classification; mlst (https://github.com/tseemann) for MLST identification from assembled sequences; SRST2 [10] for MLST identification by short-read alignment/mapping to pubMLST database (http://pubmlst.org/); ABRicate (https://github.com/tseemann) for identification of antibiotics resistance genes (ARGs) and virulence factors (VFs); BLAST [11] for sequence similarity search; snippy (https://github.com/tseemann) for single nucleotide variant (SNV) detection by short-read alignment/mapping; kWIP [12] for alignment-free, k-mer-based relatedness analysis; prokka (https://github.com/tseemann) for genome sequence annotation; Roary [13] for core- and pan-genome analysis; FastTree [14] for phylogeny tree generation; and bioconductor packages in R and/or RStudio, such as ade4 [15] for SNV principal component analysis (PCA). Interactive Tree of Life (itol, http://itol.embl.de), a web-based tool, was used for phylogenetic tree display and manipulation. Genomes of *A. baumannii* AR_0078 (NCBI accession number: CP026761) and PB364 (CP040425-CP040427) strains were used as reference for alignment/mapping. PB364 complete genome was de novo assembled from both Illumina short-read and PacBio long-read NGS followed by manually polishing. MLST definitions for SRST2 were downloaded from pubMLST. The MiniKraken database was constructed for Kraken from complete bacterial, archaeal, and viral genomes in RefSeq as of August 2016, and later updated to MiniKraken2 as of October 2017 along with Kraken2. The ARG database was integrated as of March 2017 from ResFinder [16], ARG-ANNOT [17], and CARD [18]; and VF database was from VFDB [19]. All bioinformatics data processing was conducted in the Ubuntu (v16.04) Linux platform with default settings of each algorithm except those specified in particular. The SHELL scripts used for batch data processing are provided in the Boxes of the Supplementary Materials.

## 3. Results 

**Figure 1:**
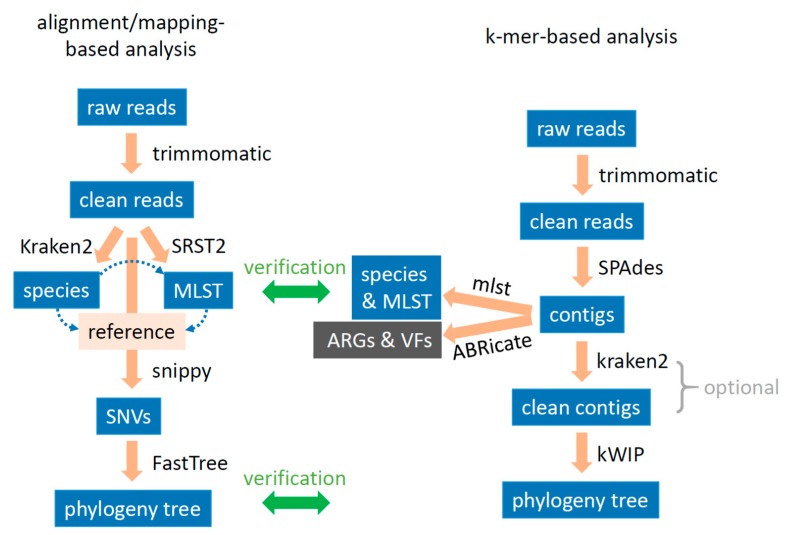
Schematic bioinformatics data processing workflows for whole-genome sequence analysis. Left: alignment/mapping-based analysis. Multi-locus sequence typing (MLST) is based on species determination, while the reference genome is selected based on both species and MLST uncovered. Right: k-mer-based analysis. Contigs cleaning is optional, mainly for those isolates with an outstanding size of the assembled “genome”. SNVs: single nucleotide variants; ARGs: antibiotic resistance genes; VFs: virulence factors.

Initially, we developed a WGS data processing pipeline based on the alignment/mapping method (Figure 1 left side). Firstly, we used the trimmomatic algorithm to trim and clean raw sequence reads. Secondly, we used Kraken classification to identify species of a single isolate and to detect if there is any mix-up or contamination in the sample. Thirdly, with the identified species, we used SRST2 to sort out MLST, since MLST is widely used to differentiate isolates in clinical laboratories. Fourthly, we chose a proper genome as reference based on the detected species and MLSTs in a population and used the snippy algorithm to identify SNVs for the characterization of each isolate. Lastly, based on their individual SNVs, we measured genetic relatedness in the bacterial population and conducted a PCA of SNVs for overview and clustering.

**Figure 2:**
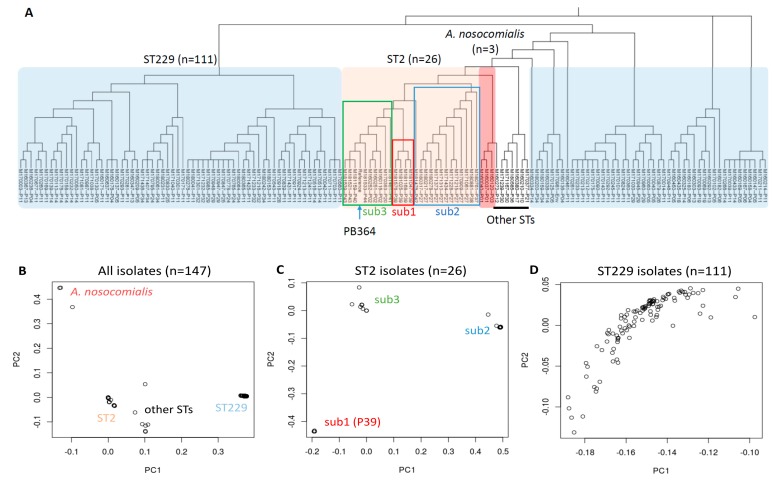
Alignment/mapping-based analysis of 147 *Acinetobacter* clinical isolates using the PB364 genome (ST2) as reference. (**A**) Phylogeny tree generated from core single nucleotide variants (SNVs) analysis. Neighbor-joining method is used, and branch length is ignored. The shadowed reference is PB364. (**B**–**D**) Overview of clinical isolates from principal component analysis. B: all 147 isolates; C: 26 ST2 isolates with PB364 excluded; and D: 111 ST229 isolates. Three main sub-clusters are identified in ST2 isolates: sub1, sub2 and sub3.

During our surveillance of multidrug-resistant pathogens from November 2016 to the end of 2018, we detected a total of 147 *A. baumannii* isolates from 34 patients and one environmental swab. The clinical demographics of these isolates are demonstrated in Supplementary Table S1. WGS was conducted for each of these isolates, reaching paired-end reads of ~1.72 million (0.53–7.86 million) and a coverage of ~65× (20×–300×) per isolate in average (Supplementary Table S2). Kraken taxonomy classification identified four isolates that had an extraordinarily low percentage (<25%) of sequence reads attributed to *A. baumannii*—three of which (M160037, M160040 and M160120) were later identified as *A. nosocomialis* by a BLAST search of their assembled contigs. MLST by SRST2 revealed that these 147 clinical isolates mainly belonged to *A. baumannii* ST229 and ST2, although 30 isolates (~20%) failed in SRST2 analysis (Supplementary Table S2). Using the *A. baumannii* PB364 genome (ST2 and local) as reference and snippy analysis, we generated a phylogeny tree of these 147 isolates based on their core SNVs (Figure 2A). A “core site” for SNVs is defined in snippy as a reference genome position presenting in all the samples in this study (https://github.com/tseemann). Using a PCA of these core SNVs, we revealed mainly four clusters in the whole population—*A. nosocomialis*, ST2, ST229, and other sequence types (STs) (Figure 2B). Sub-MLST analyses further revealed three sub-clusters in 26 ST2 isolates (Figure 2C) but no discernible sub-clusters in 111 ST229 isolates (Figure 2D).

**Figure 3:**
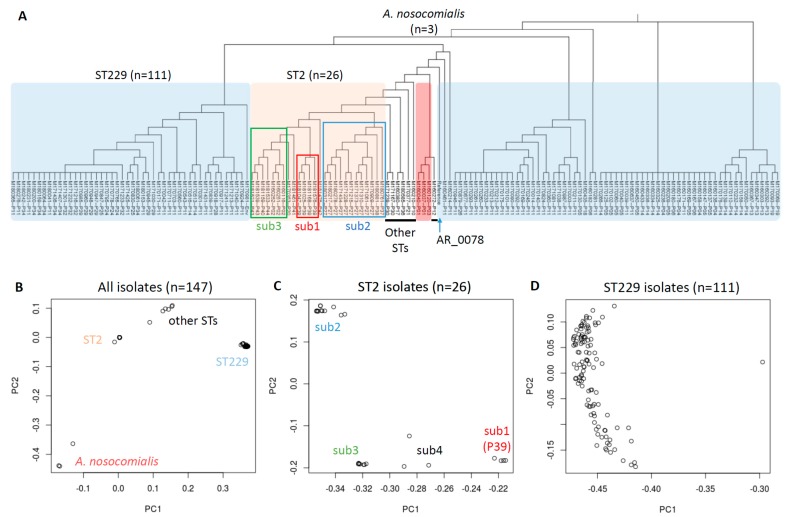
Alignment/mapping-based analysis of 147 *Acinetobacter* clinical isolates using the AR_0078 genome (ST229) as reference. (**A**) Phylogeny tree generated from core SNVs analysis. Neighbor-joining method is used, and branch length is ignored. The shadowed reference is AR_0078. (**B**–**D**) Overview of clinical isolates from principal component analysis. B: all 147 isolates; C: 26 ST2 isolates; and D: 111 ST229 isolates with AR_0078 excluded. Four main sub-clusters are identified in ST2 isolates: sub1, sub2, sub3 and sub4.

Since our majority of isolates (111 of 147) was ST229, we further used the *A. baumannii* AR_0078 genome (ST229, from CDC) as reference for a comparative analysis (Figure 3). Significant differences were found in these two core SNVs-based phylogeny trees (Figure 2A and Figure 3A), while a PCA resulted in a similar pattern of four main clusters (Figure 2B and Figure 3B). We noticed our local PB364 reference was amid our ST2 isolates in the test, whereas AR_0078 from CDC, though ST229 too, significantly differed from our collected ST229 isolates (Figure 2A and Figure 3A). In further refined sub-MLST analyses, we found four main sub-clusters in 26 ST2 isolates when AR_0078 was used as reference (Figure 3C), instead of three main sub-clusters with PB364 as reference (Figure 2C). However, we were not able to discern any sub-clusters in 111 ST229 isolates with either PB364 or AR_0078 as reference (Figure 2D and Figure 3D).

During data processing with the developed alignment/mapping pipeline, we found several drawbacks (please refer to Discussion). To circumvent the reference limitation and simplify the pipeline, we developed a k-mer-based workflow (Figure 1 right side). K-mer refers to a sub-sequence of length k within a sequence. After trimming and cleaning with trimmomatic, we employed SPAdes to de novo assemble short reads into large fragment contigs, using k-mers of 21, 33, 55, and 77 as recommended. Further, the assembled contigs were subjected to mlst algorithm for quick identification of species and MLST; and to ABRicate algorithm for quick identification of ARGs and VFs. Notably, sample mix-up or contamination can be detected by checking up the assembled “genome” size. To those with an outstanding “genome” size (defined as 10% more than the normal genome), we employed the Kraken2 taxonomy classification tool for digital clean up, using the genus level as cutoff threshold to remove contig sequences not belonging to the classified isolate. Lastly, we employed kWIP, the k-mer weighted inner product, with a k-mer set at 31 to estimate the genetic similarity of bacterial isolates in a certain population and/or (sub-)group.

**Figure 4:**
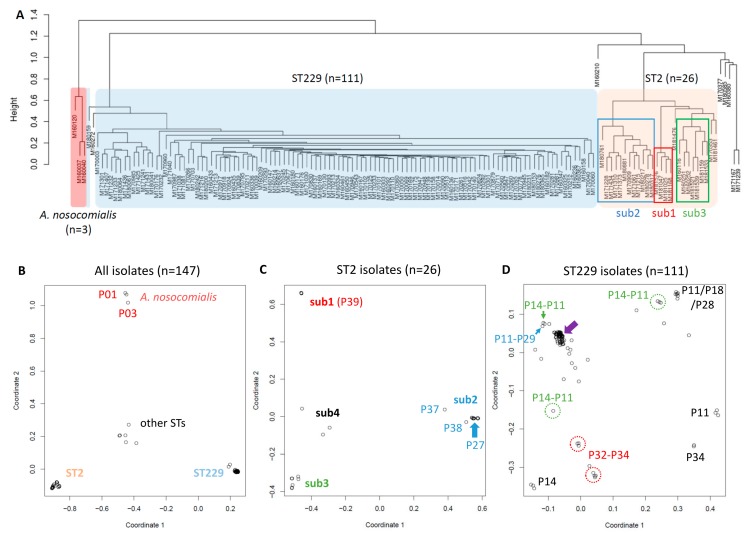

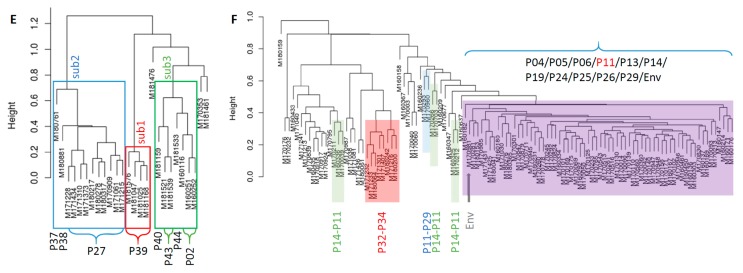
K-mer-based kWIP (k-mer weighted inner product) analysis of 147 *Acinetobacter* clinical isolates. (**A**) Phylogeny tree from hierarchical clustering using the complete linkage method. (**B**–**D**) Plots of metric multidimensional scaling (MDS). B: all 147 isolates; C: 26 ST2 isolates; and D: 111 ST229 isolates. Four main sub-clusters are identified in ST2 isolates: sub1, sub2, sub3 and sub4. Filled arrow in D shows a major sub-cluster of ST229 isolates with close relatedness. Dash-circled or arrowed are sub-clusters shared between patients as annotated aside. (**E**,**F**) Phylogeny trees of ST2 and ST229 isolates from hierarchical clustering using the complete linkage method. Three main sub-clusters are annotated as sub1, sub2 and sub3 of ST2 in E, with patients’ identities at bottom; the remaining three isolates belong to sub4. Shadowed boxes in F indicate shared sub-MLST types between patients as annotated at bottom. Isolates of the major sub-cluster in D belong to multiple patients as noted in F. The environmental isolate (Env) is arrowed grey.

We implemented this k-mer-based pipeline in the analysis of the 147 *Acinetobacter* clinical isolates, results of which are shown in Figure 4. After de novo assembly, we found eight isolates (5.4%) had an outstanding “genome” size (≥ 4.4 Mb, as we estimated *A. baumannii* genome at ~4.0 Mb), and applied Kraken2 for clean up using *Acinetobacter* genus level as cutoff (Supplementary Table S2). MLST from the assembled genome had better identification than SRST2 did, with only five isolates (3.4%) not being typed. Of these five isolates, three (M180217, M180278 and M180317) carried a SNV in fusA gene, one (M160380) was a new ST, and the other one (M160272) was mixed up with an *A. nosocomialis* isolate (species *A. baumannii*, not genus, was therefore exceptionally used as the cutoff in clean up). Notably, the three *A. nosocomialis* isolates (M160037, M160040 and M160120) were mis-recognized by the mlst algorithm as ST359 and ST71 of *A. baumannii* (Supplementary Table S2). In kWIP metric multidimensional scaling (MDS) plots, similar to a PCA, we observed significantly better clustering of all samples into four main clusters; of 26 ST2 isolates into four main sub-clusters; and of 111 ST229 isolates with a major and some other sub-clusters (Figure 4B–D), comparing to those from alignment/mapping-based method (Figure 2B–D and Figure 3B–D). The improved clustering indicated little evidence of HAI in clusters of *A. nosocomialis* and STs other than ST2 and ST229; and the refined sub-MLST clustering refuted suspected HAI of ST2 isolates (Figure 4E), among which the closest relatedness was between M181159 (patient P40, Unit H), M181521 and M181539 (patient P43, Unit F). Notably, refuting outbreaks could reduce unnecessary infection control investigation and intervention [6] and save significantly on the financial cost. However, the refined sub-MLST clustering of ST229 isolates supported the suspected transmission (Figure 4D,F). The major sub-cluster of ST229, representing the main transmission cluster, included isolates from patients P04–P06, P11, P13, P14, P19, P24–P26, and P29, and an environmental isolate (closely related to some of P11 isolates). Although they could be tracked down further by zoom-in with kWIP sub-sub-cluster analysis, we reckoned the results might be jeopardized by background noises from contaminations and/or bacterial evolution in patients. Intriguingly, based on the kWIP analysis of genomic context, we detected five sub-MLST types in patient P14 and approximately six in P11 during their hospital stay, four of which were shared between them; two sub-MLST types in P32 and three in P34, two of which were shared in both patients; additionally, two sub-MLST types shared by patients P29 and P11 (Figure 4D,F).

Recently, core- and pan-genomes have been used for the comparative analysis of multiple genomes, with multiple tools being developed [13,20,21,22]. Their biggest advantage is taking into account all functional genes existing in the pan-genome of the study. To explore its potential in bacterial relatedness analysis, we utilized prokka for annotation of all the de novo assembled genomes, followed by Roary for core- and pan-genome analysis. Results from the SPAdes-(Kraken2)-prokka-Roary pipeline are shown in Supplementary Figure S1, using Kraken2 for clean-up of isolates with mix-up/contamination as done previously in the k-mer-based protocol. We found Roary was coarse in clustering resolution, with only three sub-clusters identified in 26 ST2 isolates, similar to the result from alignment/mapping using PB364 as reference. We therefore did not go further with sub-MLST analysis.

**Figure 5:**
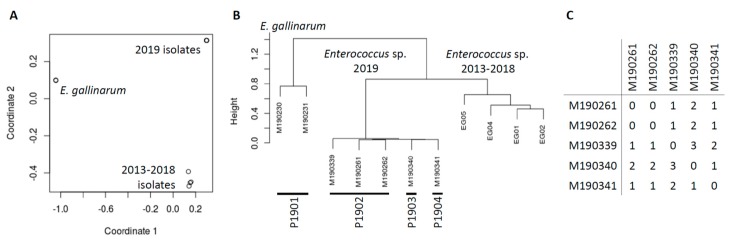
Whole-genome analysis of 11 *Enterococcus* sp. isolates. (**A**) Plot of metric multidimensional scaling (MDS) based on kWIP analysis. (**B**) Phylogeny tree from kWIP weighted metric hierarchical clustering. (**C**) Pair-wise comparisons of single nucleotide variants (SNVs) based on snippy analysis using the M190262 draft genome as reference.

The biggest advantage of using the k-mer-based workflow is that it is reference and annotation free. Moreover, we applied the de novo genomic analysis in the investigation of a suspected transmission involving five Enterococcus isolates. Although MALDI-TOF MS in the clinical laboratory indicated the five isolates collected from three patients in two service rooms were *E. gallinarum*, the de novo assembly of WGS revealed they were likely a novel *Enterococcus* sp. with no close reference genome available for alignment/mapping. For a side-to-side comparison of genetic context, we included an additional two 2019 and four previously collected (2013–2018) *E. gallinarum*-like clinical isolates. As demonstrated below, these localized and historical controls were very useful in result interpretation. Clinical demographics and WGS results of these 11 isolates are shown in Supplementary Table S3. The MDS plot and phylogeny tree of these 11 isolates are shown in Figure 5A,B, demonstrating three clearly discriminatory clusters. A BLAST search of assembled contigs uncovered that the added two 2019 isolates truly belonged to *E. gallinarum*, the four historical isolates were highly similar to *Enterococcus* sp. FDAARGOS_375 (CP023515), whereas the five HAI suspected isolates were close to *Enterococcus* sp. FDAARGOS_553 (CP033740) but with relatively low similarity, likely a novel species. All these isolates carried *vanC* operon in the chromosome, responsible for vancomycin resistance. Using FDAARGOS_553 complete genome as reference, we manually assembled a whole genome draft of isolate M190262 (CP040461-CP040462) and subsequently applied it as reference for sub-cluster snippy alignment/mapping validation. We found only 0–3 SNVs between the five clinical isolates (Figure 5C), confirming their close genomic relatedness, consistent with suspected transmission.

## 4. Discussion

WGS has been transforming microbiology, including many clinical laboratory tests for surveillance and diagnostics. Bioinformatics is key to unleashing the power and potential of WGS. Here, we developed and optimized a bioinformatics pipeline for the direct WGS analysis of bacterial isolates. We proposed a three-level data analysis, with species and MLST results to connect with current clinical laboratory tests and sub-MLST results for refined precision clustering and relatedness analysis. Our optimized k-mer-based WGS data processing workflow can rapidly and precisely estimate genomic similarity in a population, providing sequence-based evidence for precision surveillance and control of HAI, which may be applied to clinical diagnostics as well. With further improvement in automation and better integration with clinical information, the pipeline will facilitate the WGS application in clinical laboratories and make WGS a feasible tool for the research community.

During the development and comparison of our WGS data processing pipelines, the alignment/mapping-based SNV analysis demonstrated certain disadvantages. First, the whole workflow is step-by-step, from species, MLST to SNV identification, which is relatively slow and time-consuming. Second, SRST2 requires species identification and a higher depth of sequencing but has a relatively low rate (~80%) in MLST identification. Third, selection of reference genome is critical for accurate SNV detection and the followed relatedness measurement. Using one MLST reference is likely biased in the analysis of isolates with other MLSTs. A sub-MLST analysis with multiple references is thus necessary for precision results. However, finding an appropriate reference, better from a localized clone as we noted above, is troublesome and limited by its availability, whereas de novo assembling a novel genome reference is sometimes costly and time-consuming. Fourth, the alignment/mapping of short-reads has certain defects in identifying genomic structural changes, such as large fragment insertion, deletion, or inversion. Of important note, large fragment insertion/deletion in the reference genome is the main defect leading to alignment/mapping bias. Fifth, clinical isolates are sometimes mixed-up or contaminated, which will bring in significant noises or errors in MLST identification and SNV detection when short-read alignment/mapping is employed. Dealing with such mix-ups and contaminations is challenging.

There are two main reasons for the better discrimination resolution using the k-mer-based analysis workflow. One is that the kWIP clustering takes into account both SNVs and genomic structural changes (insertion, deletion, inversion etc.), with more information included than the alignment/mapping method does. The other is that the de novo genome assembly significantly normalizes the relevant weighted signals between isolates by keeping key genomic elements and reducing repetitive sequences (e.g., often, only one copy of 16S and 23S rRNA elements remained), which elevates the differential signal/noise ratio in subsequent kWIP analysis. Of additional note, using kWIP after de novo assembly significantly expedites the data processing, much faster than using kWIP directly on short-reads of each isolate, and requires relatively low sequencing depth in analysis. kWIP uses Shannon entropy, in which the weights of common and infrequent k-mers are assigned lower but non-zero weights, allowing them to contribute to the final signal [12]. Therefore, any mix-up or contamination in WGS will elevate the weighted signal, bringing in bias to the subsequent clustering and relatedness analysis, also due to the de novo assembly. Despite that such mix-up and contamination can be digitally removed by using Kraken classification, the clean-up step may also bring in bias because of the incompleteness and updating status of Kraken database. It is possible that the clean-up with Kraken mistakenly removes contig sequences truly belonging to the isolate, especially when those large mobile genetic elements (from horizontal transfer or other mechanisms) are novel to the genus and/or species. A similar scenario could also happen when setting up the minimal contig size in SPAdes de novo assembling. Moreover, it is worth mentioning that despite the low rate, mis-assembly by SPAdes assembler may jeopardize the following results of kWIP analysis, although such bias has been minimized by using the same parameters in the same pipeline. Nevertheless, we suggest a validation of the results from our k-mer-based data processing pipeline with alignment/mapping-based methods, if a suitable reference is available (Figure 1), as we demonstrated in the VRE case (Figure 5). We believe cross-examination with two distinct principles is crucial for precision outcome.

In contrast to the alignment/mapping method using SNV and kWIP method using k-mer as an analysis unit, the pan-/core-genome method uses annotated genes. More or less, the pan-/core-genome analysis ignores intergenic sequences and genomic structure. Conceptually, it is an extension of MLST and thus called cgMLST when core genes are utilized; and wgMLST, when both core and accessory genes are taken into account. As an analysis unit for refined bacterial relatedness analysis, we thus reckon SNV is too small in size and the gene is too big (or too rigid), whereas the k-mer, flexible in size with an adjustable k-mer parameter setting, is more proper and fits better.

While this manuscript was in preparation, TORMES [23], an automated and user-friendly pipeline for whole bacterial genome analysis, was published, which is capable of generating an interactive and web browser-compatible report. Regrettably, it did not include population-based k-mer relatedness analysis or digital clean-up of mix-up/contamination. Additionally, PopPUNK [24], population partitioning using nucleotide k-mers was developed for fast and flexible population analysis and clustering, which uses k-mers of variable length to calculate core and accessory distances and further define isolates, also allows interactive visualization and online report with multiple platforms. It will be of great interest to side-by-side compare results from and usage of kWIP and PopPUNK and incorporate the better one into our k-mer-based WGS data processing pipeline. With NGS becoming cheaper and more bioinformatics tools being developed, we believe proactive genomic sequencing and precision genomic surveillance will shift the practice of pathogen detection and control, and benefit both patients and health care systems. 

## Supplementary Materials

Supplementary materials can be found at https://www.mdpi.com/2076-2607/7/10/388/s1. Figure S1: Roary core- and pan-genome analysis of 147 *Acinetobacter* clinical isolates; Table S1: Clinical demographics of 147 Acinetobacter clinical isolates; Table S2: Summary of whole-genome sequencing and its bioinformatics analysis results of 147 Acinetobacter clinical isolates; Table S3: Clinical demographics and whole-genome sequencing results of 11 vancomycin-resistant enterococci isolates.
