# Supplementary material for: Optimizing a Whole-Genome Sequencing Data Processing Pipeline for Precision Surveillance of Health Care-Associated Infections
Source: Microorganisms. 2019 Sep 24;7(10):388. doi: 10.3390/microorganisms7100388 (PMC6843764; doi:10.3390/microorganisms7100388)
Supplement: Supplementary file 1 [file microorganisms-07-00388-s001.zip › SupplementaryBoxes.docx]

**Supplementary Boxes:** SHELL scripts used for batch data processing.

NOTE: All the algorithms used below are publicly available and are installed in /usr/bin/ or /usr/local/bin/, using the Ubuntu 16.04 platform. For the detail of their installations, please refer to each algorithm. Before running the scripts below, please set up the directories first and make sure that the sequence files are in .fastq.gz format, with the first “_” separating the sample ID from the remains; and that each sample ID is unique.

##################################################

# **BOX 1:** trimming and cleaning using trimmomatic #

##################################################

#!/bin/bash

threads=16

fastq_path="/path/to/fastq/files"

trimmed_path="/path/to/trimmed/folder"

cd $fastq_path

printf '%s\n' ./*.fastq.gz | cut -d_ -f1 | uniq | while read -r line; do

mkdir $trimmed_path/"$line";

cd $trimmed_path/"$line";

TrimmomaticPE -threads $threads -phred33 $fastq_path/"$line"*_R1*.fastq.gz $fastq_path/"$line"*_R2*.fastq.gz "$line"_R1.fq.gz /dev/null "$line"_R2.fq.gz /dev/null ILLUMINACLIP:/usr/share/trimmomatic/NexteraPE-PE.fa:2:30:10 LEADING:3 TRAILING:3 SLIDINGWINDOW:4:15 MINLEN:36;

done

###############################################################

# **BOX 2:** de novo assembly using SPAdes + identification of #

# species, MLST, ARGs and VFs using mlst & ABRicate #

# **Note:** abricate database is a merge of ResFinder, ARG-ANNOT, #

# and CARD databases; and vfdb database is from VFDB. #

###############################################################

#!/bin/bash

threads=16

trimmed_path="/path/to/trimmed/fastq/files"

temp_path="/path/to/temporary/folder"

assembled_path="/path/to/assembled/folder"

cd $trimmed_path

for id in $(ls -d *); do

cd $id;

python3.5 /usr/local/bin/spades.py -k 21,33,55,77 --careful -1 "$id"_R1.fq.gz -2 "$id"_R2.fq.gz -o $temp_path/spades;

mv $temp_path/spades/contigs.fasta ./"$id"_contigs.fa;

mv $temp_path/spades/spades.log ./"$id"_spades.log;

rm -f -v -r $temp_path/spades;

mlst "$id"_contigs.fa > "$id"_mlst_assembly.tab;

abricate --db abricate "$id"_contigs.fa > "$id"_arg.tab;

abricate --db vfdb "$id"_contigs.fa > "$id"_vfdb.tab;

cd ..;

mv $id $assembled_path;

done

######################################################################

# **BOX 3:** taxonomy classification of contigs using kraken2 (optional) #

######################################################################

#!/bin/bash

list="clean.list"

thread=16

base_path="/path/to/folder/of/isolates"

minikraken_database="/path/to/kraken2_database/Mini_Kraken2_10182017"

cd $base_path;

cat $list | while read id; do

cd "$id";

kraken2 --db $minikraken_database --threads $thread --report "$id"_contigs.kreport2 "$id"_contigs.fa > "$id"_contigs.kraken2;

cut -f2,3 "$id"_contigs.kraken2 > "$id"_contigs.krona2.in;

ktImportTaxonomy "$id"_contigs.krona2.in -o "$id"_contigs.krona2.html;

cd ..;

done

################################################################################

# **BOX 4:** digital cleaning of mix-up/contamination by extracting contigs needed #

# **Note:** open the "$id"_contigs.krona2.html file in web browser for viewing; #

# find the particular contigs belonging to the isolate and use the genus level #

# as cutoff for extraction; copy and paste these contig IDs into #

# "$id"_clean.list file for each isolate to be cleaned. #

################################################################################

#!/bin/bash

list="clean.list"

thread=16

base_path="/path/to/folder/of/isolates"

cd $base_path;

cat $list | while read id; do

cd "$id";

mv "$id"_contigs.fa "$id"_contigs_ori.fa;

cut -c 1- "$id"_clean.list | xargs -n 1 samtools faidx "$id"_contigs_ori.fa > "$id"_contigs.fa;

cd ..;

done

##########################################

# **BOX 5:** relatedness analysis using kWIP #

##########################################

#!/bin/bash

threads=16

list="/list/of/file/for/input"

work_path="/path/to/folder/for/kWIP/analysis"

base_path="/path/to/folder/of/isolates"

hash_path="/path/to/folder/for/hashes"

cd $base_path

cat $list | while read id; do

khmer load-into-counting.py -N 1 -x 1e9 -k 31 -b -T $threads -f -s tsv $hash_path/${id}.ct.gz $fastq_path/${id}/${id}_contigs.fa

done

cd $work_path

kwip -t 2 -k Name_of_Output.kern -d Name_of_Output.dist $hash_path/*.ct.gz

Rscript /path/to/kWIP/util/img.R Name_of_Output

###############################################################

# **BOX 6:** taxonomy classification of short-reads using kraken2 #

###############################################################

#!/bin/bash

threads=16

trimmed_path="/path/to/trimmed/fastq/files"

krakened_path="/path/to/krakened/files"

minikraken_db="/path/to/kraken2_database/Mini_Kraken2_10182017"

cd $trimmed_path

for id in $(ls -d *); do

cd $id;

kraken2 --db $minikraken_db --threads $threads --paired --gzip-compressed --report "$id".kreport "$id"_R1.fq.gz "$id"_R2.fq.gz > "$id".kraken

awk '$4=="S"' "$id".kreport | cut -f1,6 | sort -nr > "$id".species;

cut -f2,3 "$id".kraken > "$id".krona.in;

ktImportTaxonomy "$id".krona.in -o "$id".krona.html;

cd ..;

mv $id $krakened_path/;

done

####################################

# **BOX 7:** MLST analysis using SRST2 #

####################################

#!/bin/bash

threads=16

temp_path="/path/to/temporary/folder"

base_path="/path/to/folder/of/isolates"

srst2db_path="/path/to/srst2_database/folder"

cd $base_path

for id in $(ls -d *); do

cd $id;

mv "$id"_R1.fq.gz "$id"_1.fastq.gz;

mv "$id"_R2.fq.gz "$id"_2.fastq.gz;

mkdir $temp_path/"$id";

srst2 --threads $threads --input_pe "$id"_1.fastq.gz "$id"_2.fastq.gz --output $temp_path/$id/"$id" --log --mlst_db $srst2db_path/Acinetobacter_baumannii#2.fasta --mlst_definitions $srst2db_path/abaumannii_2.txt --mlst_delimiter '-' --gene_db $srst2db_path/ARGannot.r1.fasta;

mv "$id"_1.fastq.gz "$id"_R1.fq.gz;

mv "$id"_2.fastq.gz "$id"_R2.fq.gz;

mv $temp_path/$id/"$id"__mlst__Acinetobacter_baumannii#2__results.txt ./"$id"_mlst_srst2.txt;

mv $temp_path/$id/"$id"__fullgenes__ARGannot.r1__results.txt ./"$id"_arg_srst2.txt;

mv $temp_path/$id/"$id".log ./"$id"_srst2.log;

rm -f -v -r $temp_path/$id;

cd ..;

done

###############################################################################

# **BOX 8:** SNV-based phylogeny analysis using snippy, snippy-core, and FastTree #

###############################################################################

#!/bin/bash

base_path="/path/to/folder/of/isolates"

reference="/path/to/file/of/reference"

snps_output="/path/to/folder/of/SNV_output"

snippy_path="/path/to/folder/of/snippy_results"

cd $base_path

for id in $(ls -d *); do

cd $id;

snippy --cpus 16 --outdir $snps_output/"$id" --ref $reference --R1 "$id"_R1.fq.gz --R2 "$id"_R2.fq.gz;

printf '%s ' $snps_output/"$id";

cd ..;

done > $snippy_path/snp_files

cd $snippy_path

snippy-core --prefix core $(cat snp_files)

FastTree -nt core.aln > core.nwk

perl /usr/local/bin/afa-pairwise.pl core.aln > distance_snippy.tab

#########################################

# **BOX 9:** genome annotation using prokka #

#########################################

#!/bin/bash

base_path="/path/to/folder/of/isolates"

list_file="/home/genomics/Desktop/temp/list"

roary_path="/path/to/folder/of/roary"

threads=16

cd $base_path

for id in $(cat $list_file); do

cd $id;

prokka --outdir "$id".prokka --cpus $threads --evalue 1e-10 --gram neg --kingdom Bacteria --genus Acinetobacter --strain "$id" --centre NYMC --prefix "$id" --addgenes "$id"_contigs.fa;

printf '%s ' "$PWD"/"$id".prokka/"$id".gff;

cd ..;

done > $roary_path/gff_files

#####################################################

# **BOX 10:** core- and pan-genome analysis using roary #

#####################################################

#!/bin/bash

threads=16

roary_path="/path/to/folder/of/roary"

cd $roary_path

roary -f roary_results -e -n -v $(cat $roary_path/gff_files)

cd roary_results

FastTree -nt -gtr core_gene_alignment.aln > core_gene_alignment.nwk

python /usr/local/bin/roary_plots.py core_gene_alignment.nwk gene_presence_absence.csv

####################################

# **BOX 11:** PCA of SNVs in R/RStudio #

####################################

setwd("/path/to/folder/for/analysis")

library("dplyr", "ade4")

samples.id <- read.csv("sample.id", header = F)

snps.data <- read.csv("snps.vcf", header = F)

dim(snps.data)

snps.data <- dplyr::select(snps.data, 1:147)

colnames(snps.data) <- samples.id$V1

pca <- dudi.pca(snps.data, scale = F)

s.label(pca$co, sub="PCA - PC 1 & 2")

add.scatter.eig(pca$eig, 4,1,2, ratio=0.3, posi="bottomright")

D <- dist(pca$co[1,2])^2

clust <- hclust(D, method="complete")

plot(clust, main = "Clustering (complete linkage) based on the first 2 PCs", cex=.4)

##################################

# **BOX 12:** summary of WGS results #

##################################

#!/bin/bash

base_path="/path/to/folder/of/isolates"

summary_path="/path/to/folder/of/summary"

cd $base_path

for id in $(ls -d *); do

cd $id;

printf "$id";

printf "\t";

zcat "$id"_R1.fq.gz | printf $((`wc -l`/4));

printf "\t";

grep -v ">" "$id"_contigs.fa | wc | awk '{printf $3-$1}';

printf "\t";

printf "$(grep -c "^>" "$id"_contigs.fa)";

printf "\t";

printf "$(awk 'NR==1' "$id".species | cut -f 1-2)";

printf "\t";

printf "$(cat "$id"_mlst_assembly.tab | cut -f 2-10)";

printf "\t";

printf "$(awk 'NR==2' "$id"_mlst_srst2.txt | cut -f 2-13)";

printf "\n";

cd ..;

done > $summary_path/"summary"
