# Supplementary material for: Optimizing a Whole-Genome Sequencing Data Processing Pipeline for Precision Surveillance of Health Care-Associated Infections
Source: Microorganisms. 2019 Sep 24;7(10):388. doi: 10.3390/microorganisms7100388 (PMC6843764; doi:10.3390/microorganisms7100388)
Supplement: Supplementary file 1 [file microorganisms-07-00388-s001.zip › SupplementaryFigureS1.docx]

**
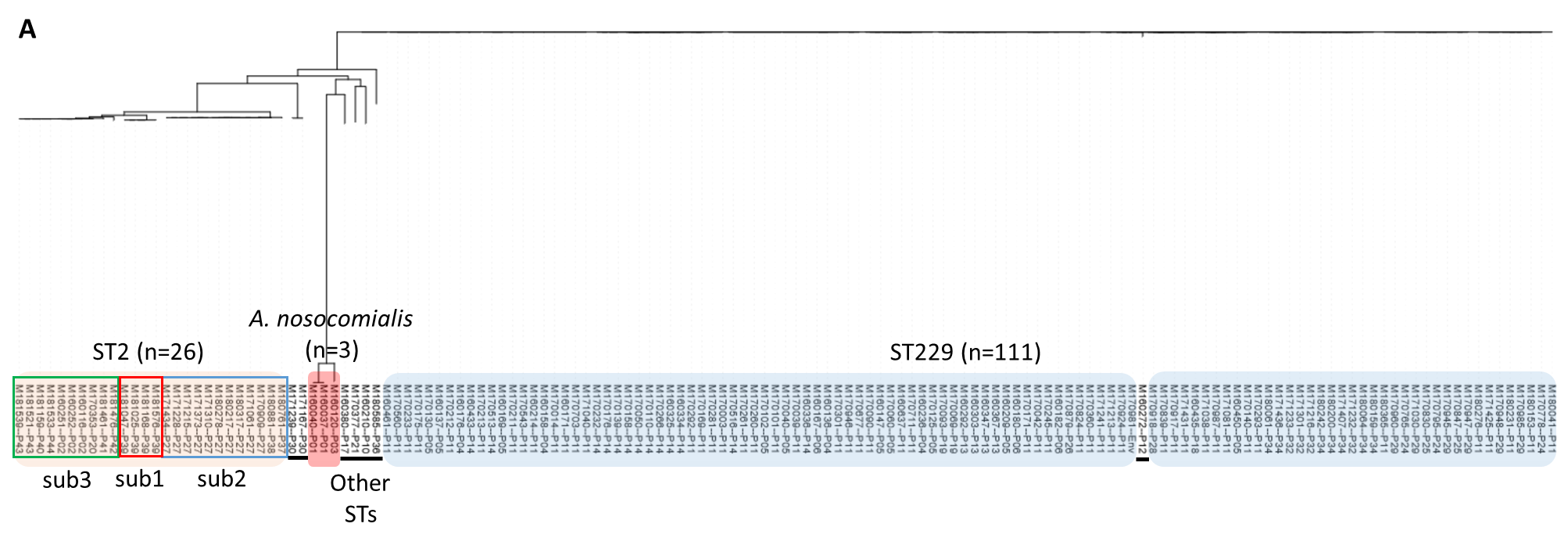
**

**
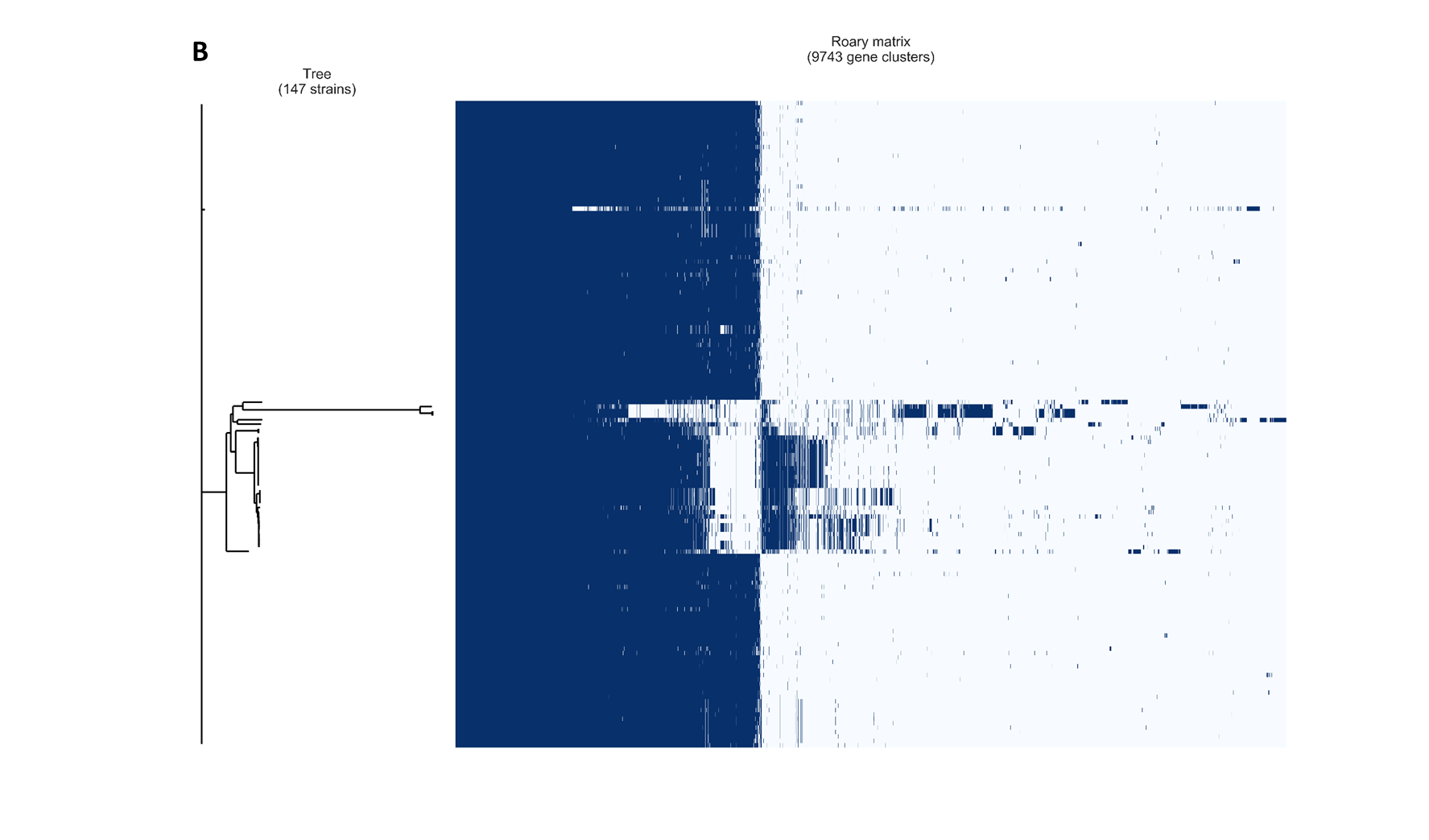
**

**Supplementary Figure S1.** Roary core- and pan-genome analysis of 147 *Acinetobacter* clinical isolates. A) Phylogeny tree from Roary core- and pan-genome analysis. Four main clusters are identified: 3 *A. nosocomialis* isolates; 26 ST2 isolates; 111 ST229 isolates; and other *A.* *baumannii* isolates with other STs (underlined). Only three sub-clusters are identified in ST2 isolates: sub1, sub2 and sub3. B) Roary matrix plot of 9743 gene clusters identified from the 147 clinical isolates.
